# A data-centric way to improve entity linking in knowledge-based question answering

**DOI:** 10.7717/peerj-cs.1233

**Published:** 2023-02-09

**Authors:** Shuo Liu, Gang Zhou, Yi Xia, Hao Wu, Zhufeng Li

**Affiliations:** Information Engineering University, Zhengzhou, Henan, China

**Keywords:** Entity linking, Negative sampling, Natural language processing, Knowledge-based question answering

## Abstract

Entity linking in knowledge-based question answering (KBQA) is intended to construct a mapping relation between a mention in a natural language question and an entity in the knowledge base. Most research in entity linking focuses on long text, but entity linking in open domain KBQA is more concerned with short text. Many recent models have tried to extract the features of raw data by adjusting the neural network structure. However, the models only perform well with several datasets. We therefore concentrate on the data rather than the model itself and created a model DME (Domain information Mining and Explicit expressing) to extract domain information from short text and append it to the data. The entity linking model will be enhanced by training with DME-processed data. Besides, we also developed a novel negative sampling approach to make the model more robust. We conducted experiments using the large Chinese open source benchmark KgCLUE to assess model performance with DME-processed data. The experiments showed that our approach can improve entity linking in the baseline models without the need to change their structure and our approach is demonstrably transferable to other datasets.

## Introduction

**Figure 1:**
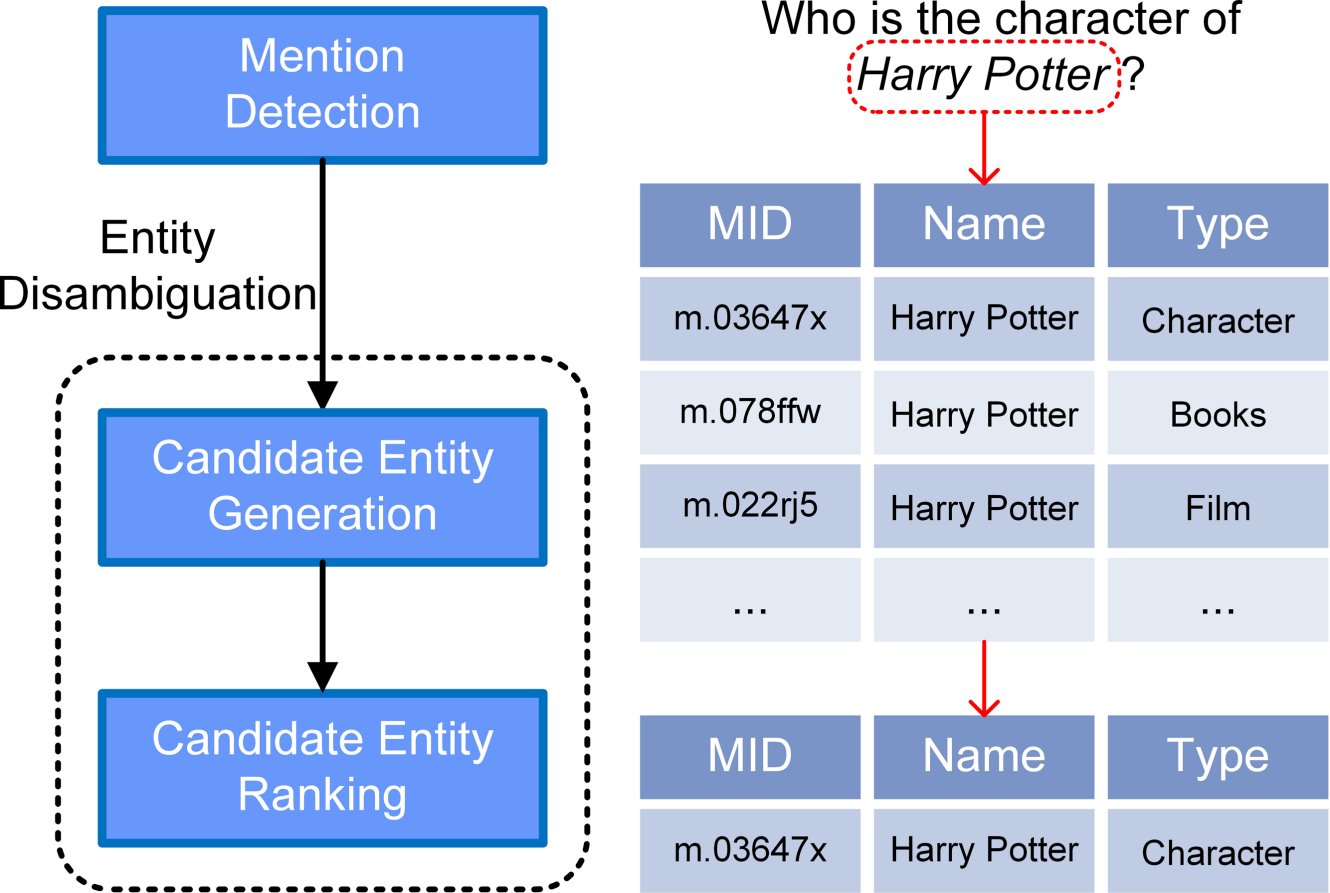
A specific example of our pipeline model. The input question is “Who is the character of Harry Potter?” Typical results of the query are shown. And our model successfully linked the mention “Harry Potter” to the correct entity node of the KB.

In order to make use of ever-increasing quantities of data, many knowledge bases (KBs), such as Freebase (Bollacker et al., 2008) and DBpedia (Auer et al., 2007), have collected natural language network data which they formatted as triples $ \left( h,r,t \right) $, where *h* and *t* are subject and object entities, and *r* is the relation between them. Regular internet users may lack of the technical skills required to query such datasets easily. KBQA aims to answer the natural language question revolves around the KB, which provide a portable way to access KB for normal users (Huang et al., 2021). In KBQA, entity linking (EL) is an important approach to connecting natural language queries to formalized KBs and is usually considered to be the first step in creating a KBQA system. Table 1 shows the functions of each subtask and Fig. 1 shows the pipeline of such a tripartite EL system. The *mention* (Schindler et al., 2022) of an entity, in the remainder of the article, is the set of representations in natural language that include the phrase identifying the entity.

**Table 1:** Definition of the subtasks.

Subtask	Definition
Mention Detection	Identify entity mention from a question.
Candidate Generation	Produce a set of candidate entities in KG from the ambiguous entity mention
Entity Ranking	Rank candidate entities according to the mention-entity correspondence estimation

In recent research, there has been a tendency to transform the mention detection task into a sequence recognition task, and this technique has resulted in excellent performance of recurrent neural networks models, such as LSTM-CRF (Lample et al., 2016). EL concentrates on disambiguating entities, both candidate entity generation and candidate entity ranking are based on similarity. To improve EL, we determined it was necessary to better represent mention and entity. The semantics of a *mention* is usually influenced by the context, so polysemy is an extremely common phenomenon in natural language. Representing the mentions and entities in a multi-dimensional way is significant in entity disambiguation. Recent research in short text EL tend to enhance the presentation of mentions and entities through some external information such as the context of the mentions and the neighboring relations of the target entity. However, this approach has two drawbacks. Firstly, the relevant work has usually been model-centric and has tended to improve the model only using several datasets rather than more generally. Secondly, most Chinese KBs are immature and therefore provide only limited information, making it necessary to somehow incorporate additional information to improve the performance of the EL model. Moreover, generating negative samples is necessary for training. Conventional negative sampling is usually based on random or normal distributions, but a model cannot learn enough valuable information due to the poor fitting provided by a straightforward statistical method.

We therefore developed a model, DME (Domain information Mining and Explicit expressing), to better representing mentions without changing the model structure. To improve the quality of negative samples, our approach to negative sampling combines surface form with semantic information of the entities.

In summary, the main contributions of this study are as follows.

(1) We increased the robustness of EL in the model without changing the model structure by designing a data-centric model, DME, to mine domain information for short text.

(2) We developed an innovative negative sampling approach that generates high quality negative samples by considering both surface form and semantic information.

(3) We performed experiments using KgCLUE and NLPCC2016 and thus demonstrated our methods are effective and adaptable.

## Related Work

Sevgili et al. (2022) and Shen, Wang & Han (2015) have conducted a detailed survey about the approaches of entity linking. Much research into entity linking is concerned with entity disambiguation, in which the key issue is how best to represent the semantics of the mention and the entity and how then to rank candidate entities by semantic distance. To increase efficiency, in terms of minimizing computing time, an inexact matching may be determined that can be prioritized over deep semantic matching, which allows entity disambiguation to be divided into processes of candidate entity generation and entity ranking.

### Candidate entities generation

One approach is to match surface forms, which generates a candidate entity list by matching the surface forms between mention and entity. Many heuristics, such as Levenshtein distance (Le & Titov, 2019), n-grams (Moreno et al., 2017) and Word2vec (Zwicklbauer, Seifert & Granitzer, 2016), are used for embedding and matching the surface form of mention and entity. An alternative approach is to build an entity-mention dictionary that is expanded with aliases. Most aliases are extracted by KG metadata, such as entity pages, redirect pages, disambiguation pages and hyperlinks in Wikipedia articles (Zwicklbauer, Seifert & Granitzer, 2016; Fang et al., 2019).

### Candidate entities ranking

Research to date has been primarily concerned with ranking candidate entities rather than inexact matching. The semantic matching model is optimized to capture the deeper semantics of both mention and entity. The two principal techniques used are context-mention encoding and entity encoding.

Context–mention encoding is intended to encode the mention with information captured from the context. The usual approach is to construct a dense contextualized vector that represents the mention. An earlier approach was to encode mentions using a convolutional neural network (Francis-Landau, Durrett & Klein, 2016). However, the mainstream approach now is to use recurrent neural networks with self-attention (Vaswani et al., 2017). Some researchers have used LSTM to build a recurrent neural network to improve representation (Fang et al., 2019; Le & Titov, 2019). For example, Sil et al. (2018) encoded mention contexts using LSTM and passed the result to a coreference chain and adjusted the representation using a tensor network. Eshel et al. (2017) modified LSTM-GRU by incorporating an attention mechanism into the encoder. Attention neural network is widely used in current encoding approaches. The entity linking model proposed in (Logeswaran et al., 2019; Peters et al., 2019; Chen et al., 2020; Wu et al., 2020) exploited pre-trained BERT to capture as many multidimensional features as possible to improve mention and entity encoding.

The second approach, entity encoding, is intended to capture deep semantic information and generate a distributed vector representation for each candidate entity. In earlier research, the entity representation space was populated with unstructured texts using algorithms, such as Word2vec, that produced co-occurrence statistics (Zwicklbauer, Seifert & Granitzer, 2016; Moreno et al., 2017). Recent work has used pretrained language models (PLMs), of which BERT (Devlin et al., 2019) is representative, to encode entities (Nie et al., 2018; Mulang’ et al., 2020). For example, Logeswaran et al. (2019) and Wu et al. (2020) trained BERT using entity description pages from Wikipedia to form a supplementary representation of external information. Yamada et al. (2019) developed an entity disambiguation model which was trained using a novel masked entity prediction task. The model was trained by predicting randomly masked entities in entity-annotated texts from Wikipedia.

In addition to the approaches of encoding, some studies have improved the effectiveness of model training by enhancing the quality of training samples. To better training of the model, Rao, He & Lin (2016) exploited interactions in triplet inputs over the question paired with positive and negative examples. For the same purpose, Zhang et al. (2019) proposed a GAN-based methods, NSCaching, to sampling negative triplets from a KB.

Most of the existing approaches are modifications of existing models, which work better on certain data but are less adaptable. It means that they are not suitable for other datasets of the same task. In contrast to these approaches, our proposed DME model focuses on data-centric augmentation, and this model can simply but effectively improve the quality of text representation while being applicable to the vast majority datasets.

### Task definition

A popular implementation of EL in KBQA is to use a pipeline consisting of two modules: *mention detection* and *entity disambiguation*. However, the huge volume of data in a KB will create a large search space when calculating mention–entity similarity. *Entity disambiguation* thus can be split into two subtasks, including *candidate generation* and *entity ranking* (Sevgili et al., 2022). $G= \left( V,E \right) $ represents the entities and relations in a KB, where $V= \left\{ {v}_{1},{v}_{2},\ldots ,{v}_{n} \right\} $ contains all of the entities, $Q= \left\{ {q}_{1},{q}_{2},\ldots ,{q}_{n} \right\} $ and $M= \left\{ {m}_{1},{m}_{2},\ldots ,{m}_{n} \right\} $ are respectively the set of questions and the set of mentions that have been extracted from the questions. We can then describe the entity linking as: Given a set of entities *V* and a set of mentions *M* that are contained in a set of questions *Q*, entity linking model aims to link the mention *m* ∈ *M* that appears in a question to the entity node *e* ∈ *V* correctly. The task can be further formulated as:

(1) \begin{eqnarray*}\hat {a}=\mathit{arg~ max}_{e\in V}{P}_{r} \left( e \left\vert G,Q \right. \right) \end{eqnarray*}

where $\mathit{Pr} \left( e \left\vert G,Q \right. \right) $ is the probability of entity e being the correct linking result of the question *Q*. The target KB normally contains millions of entities, which means that directly modeling $\mathit{Pr} \left( e \left\vert G,Q \right. \right) $ is computationally intensive. One line of research forms entity linking as a *semantic matching* task, aims to finds a suitable entity within KB that is similar to the *mention* in the question. Following this direction, we divided the entity linking into three steps: (1) Recognize the mention *m*from the question *q* ∈ *Q*, where *m* is the sub-string of *q*; (2) Extract the candidate entities $\overline{v}$ that match mention *m* andconstruct a set of candidate entities $\overline{V}= \left\{ {\overline{v}}_{p},{\overline{v}}_{p+1},\ldots ,{\overline{v}}_{q} \right\} ,\overline{V}\subseteq V$; (3) Rank the candidate entities $\overline{V}$ to obtain the result *v*. The model can be factorized as:

(2) \begin{eqnarray*}\begin{array}{@{}l@{}} \displaystyle {P}_{r} \left( e \left\vert G,Q \right. \right) ={P}_{r} \left( m,\overline{V},v \left\vert G,Q \right. \right) \\ \displaystyle ={P}_{m} \left( m \left\vert G,Q \right. \right) \cdot {P}_{\overline{V}} \left( \overline{V} \left\vert m,G,Q \right. \right) \cdot {P}_{v} \left( v \left\vert \overline{V},m,G,Q \right. \right) \end{array}\end{eqnarray*}

where ${P}_{m} \left( m \left\vert G,Q \right. \right) $ is the mention detection model, ${P}_{\overline{V}} \left( \overline{V} \left\vert m,G,Q \right. \right) $ is the model of candidate entity generation, and ${P}_{v} \left( v \left\vert \overline{V},m,G,Q \right. \right) $ is a component for candidate entity ranking.

## Method

We used a conventional approach for mention detection model ${P}_{m} \left( m \left\vert G,Q \right. \right) $ and candidate entity generation model ${P}_{\overline{V}} \left( \overline{V} \left\vert m,G,Q \right. \right) $. And the central goal of our research was to use the DME model to improve the performance of the candidate entity ranking model ${P}_{v} \left( v \left\vert \overline{V},m,G,Q \right. \right) $.

### Mention detection and candidate entities generation

The mention detection model ${P}_{m} \left( m \left\vert G,Q \right. \right) $ detects the mention span within a question. Most recent approaches depend on named entity recognition (NER), which performs extremely well in mention detection. We considered mention detection to be a sequence labeling task and created a mention detection model using BERT and CRF.

We first transformed the question *q* ∈ *Q* as a BIO-labeled sequence, where *B* and *I* represent the start and inner of a mention span, and *O* labels the superfluous part of *q*. We then used BERT and CRF to model the labeled sequence.

When the mention contained in the question had been obtained, we used ${P}_{\overline{V}} \left( \overline{V} \left\vert m,G,Q \right. \right) $ to generate a set of candidate entities. Conventional approaches to candidate entity generation are mainly based on string comparison between the surface form of the mention entity and the name of the entity existing in a KB. We created a semantic space using a pretrained Word2vec algorithm and then encoded the mentions and all entities in the KB for approximate similarity matching. We finally ranked the entities by the similarity score and returned the top-*k* entities as the list of candidate entities. The *k* is a variable and we found the best recall ratio when *k* = 70.

### Candidate entities ranking

The last part of our entity linking model was to rank the candidate entities using the model ${P}_{v} \left( v \left\vert \overline{V},m,G,Q \right. \right) $, which sorts candidate entities by deep semantic matching. It is essential when mining the deeper semantics of mentions and entities to increase the dimensions of the representations. A conventional approach is to learn more features by adjusting the structure of a model and varying its parameters. This approach requires researchers to prepare massive quantities of data to train models and to expect the deep neural network to automatically capture the inexplicable features. However, for existing giant models that had been derived from BERT, some hard problems demonstrated unexpected weaknesses in this approach when being used in downstream tasks. These weaknesses included elementary errors when treating the datasets in actual conditions and magnification of bias embedded in everyday human-originated questions. In short, improvements resulted in decreasing marginal benefits after the PLMs had been developed to a particular stage. Researchers are becoming increasingly aware of the importance of the quality of data. Ng (2021) suggested that research into artificial intelligence should change its approach from model-centric to data-centric. Also, Lu et al. (2022) recognized that keywords can better represent a sentence than use all information within a sentence. In our research, we found that the context of a mention and the relations that surround an entity can carry much information concerning the domain. For example, in the question “Who directed Game of Thrones?”, the verb “direct” indicates that this question may be related to a film or television work. In the one-hop relations of the entity “Game of Thrones”, “Release time”, “Producer” and “Screenwriter” also indicate that the entity may be connected to a film or television work. These insights led us to realize that domain keywords are vital in entity linking. We devised a lightweight and transferable method of finding domain keywords by feature engineering and improve the performance of entity linking without modifying the structure of the model.

**Figure 2:**
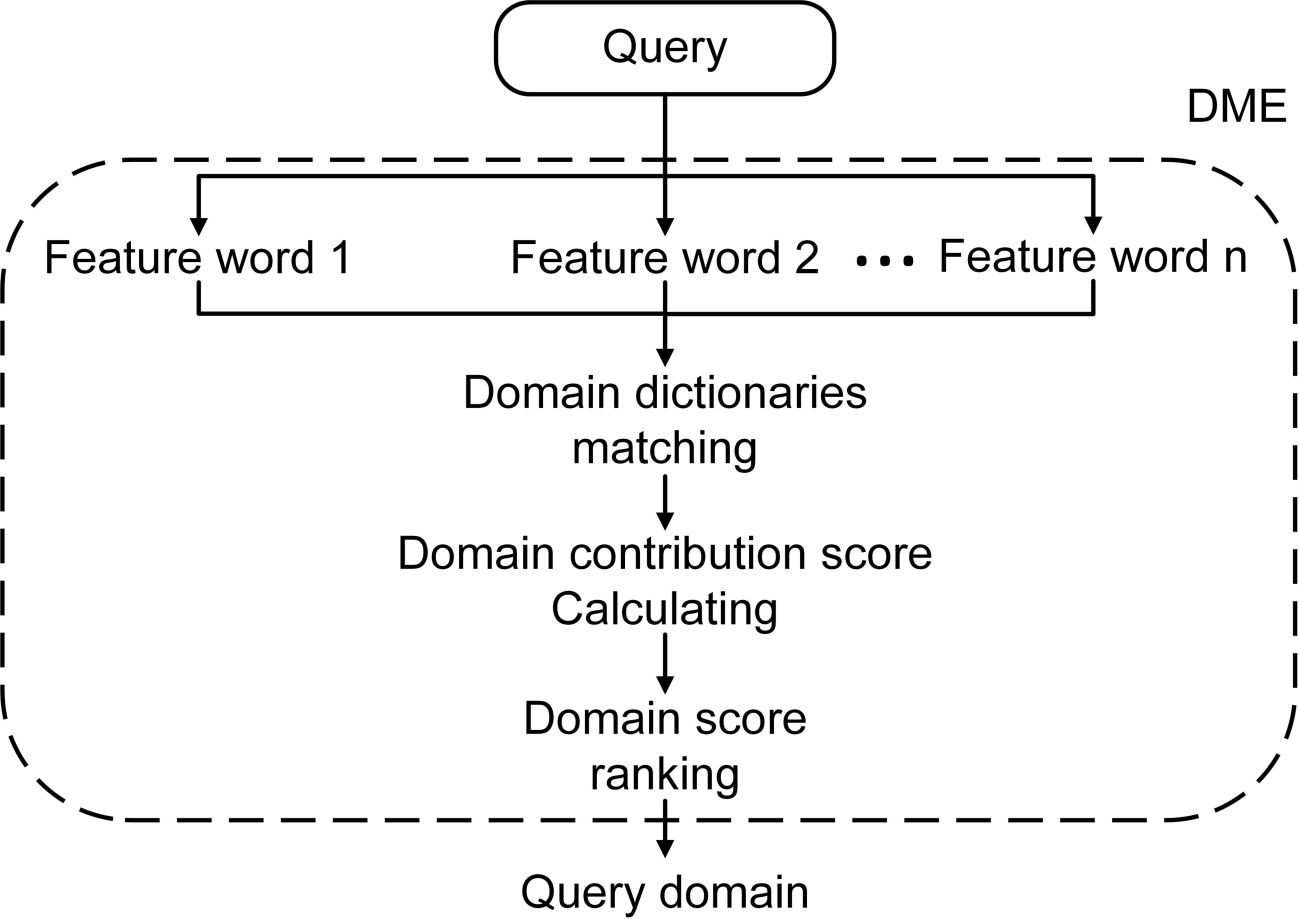
The overall structure of DME.

In this way, we developed the DME model to mine and explicitly represent the implicit domain information concealed in the text. Figure 2 shows the overall structure of DME. In detail, we used $A= \left\{ {a}_{1},{a}_{2},\cdots ,{a}_{n} \right\} $ to represent the feature words that consist of a question’s segments or an entity node with surrounding relations. $P \left( {B}_{i} \left\vert {a}_{j} \right. \right) $ represents the implicit domain indication inherent in feature term *a*_*j*_in domain *B*_*i*_. To highlighting frequently used words, we modeled the occurrence frequency with the parameter popularity *p*_*a*_*j*__:

(3) \begin{eqnarray*}{p}_{{a}_{j}}= \frac{df \left( {a}_{j} \right) }{\sum _{i=1}^{n}df \left( {a}_{i} \right) } \end{eqnarray*}

where the dictionary frequency $df \left( t \right) $ represents the number of domain directories that contains the feature term *t*. Then DME can be entirely represented as:

(4) \begin{eqnarray*}F \left( A \right) =select{S}_{{B}_{i}}=select \left( \sum _{j=1}^{n}{p}_{{a}_{j}}\cdot P \left( {B}_{i} \left\vert {a}_{j} \right. \right) \right) \end{eqnarray*}

where *S*_*B*_*i*__ represents the different domain scores of *A*, and *Select* is the selection method we developed to process the real world’s occurrence of a text being related to many different domains. We first sort *S*_*B*_*i*__ and obtain the standard deviation *σ* and then calculate the difference in score between the top-2, *s*_1_ = *S*_*B*_1__ − *S*_*B*_2__. If *s*_1_ > *σ*, then *B*_1_ will be returned as the result of $F \left( A \right) $. If *s*_1_ ≤ *σ*, both *B*_1_ and *B*_2_ will be regard as the result; *S*_*B*_2__ and *S*_*B*_3__ are then compared in the same manner.

Careful consideration needs to be given to the division of domains, as fewer domain divisions would make the distinction insufficient, while too many would make some words difficult to classify. We therefore learned from the Universal Decimal Classification (UDC) and some of its subsequent classification criteria (McIlwaine, 1997). The encyclopedia knowledge is therefore divided into nine domains. Table 2 gives examples of typical subdomains and the number of keywords for each branch. We constructed nine domain dictionaries based on millions of domain keywords.

**Table 2:** Domain dictionary.

**Domain**	**Sub-domains**	**Number**
Nature	Organism, Natural resources, Astronomical phenomena	152,000
Culture	History, Literature, Historical personages, Religion	235,600
Daily	Diet, Traffic, Tourism, Entertainment	46,000
Social	Law, Organizations, Media, Charities	103,000
Technology	Computer, Medical and Vehicle technology	200,000
Art	Painting, Music, Opera and theatre, Movies and TV, Architecture	22,280
Sport	Series competitions, Electronic sports, Team names	23,000
Politics	Military affairs, Administrative divisions, Diplomacy	131,000
Economy	Enterprise, Brands, Stock and funds, Insurance	54,000

Using Bayes’ theorem, the local domain contribution $P \left( {B}_{i} \left\vert {a}_{j} \right. \right) $can be modeled as:

(5) \begin{eqnarray*}P \left( {B}_{i} \left\vert {a}_{j} \right. \right) = \frac{P \left( {B}_{i} \right) P \left( {a}_{j} \left\vert {B}_{i} \right. \right) }{P \left( {a}_{j} \right) } \propto P \left( {B}_{i} \right) P \left( {a}_{j} \left\vert {B}_{i} \right. \right) \end{eqnarray*}

where $P \left( {a}_{j} \right) $ is the probability of a feature term occurrence, which can be treated as a constant. The probability of a randomly selected domain $P \left( {B}_{i} \right) $ is a prior probability:

(6) \begin{eqnarray*}P \left( {B}_{i} \right) = \frac{\log \nolimits ~Count \left( {B}_{i} \right) }{\sum _{i=1}^{9}\log \nolimits ~Count \left( {B}_{i} \right) } .\end{eqnarray*}

Since contribution is a relative concept that needs to be normalized later, it is not necessary to calculate the probability. We adapt the calculation in a uniform scale, which also improve the operational efficiency. We thus replaced $P \left( {a}_{j} \left\vert {B}_{i} \right. \right) $ in (5) with $C \left( {\mathrm{a}}_{j},{B}_{i} \right) $, which represents the contribution of feature term *a*_*j*_ to domain *B*_*i*_:

(7) \begin{eqnarray*}C \left( {\mathrm{a}}_{j},{B}_{i} \right) = \frac{1}{df \left( {a}_{j} \right) } .\end{eqnarray*}

**Figure 3:**
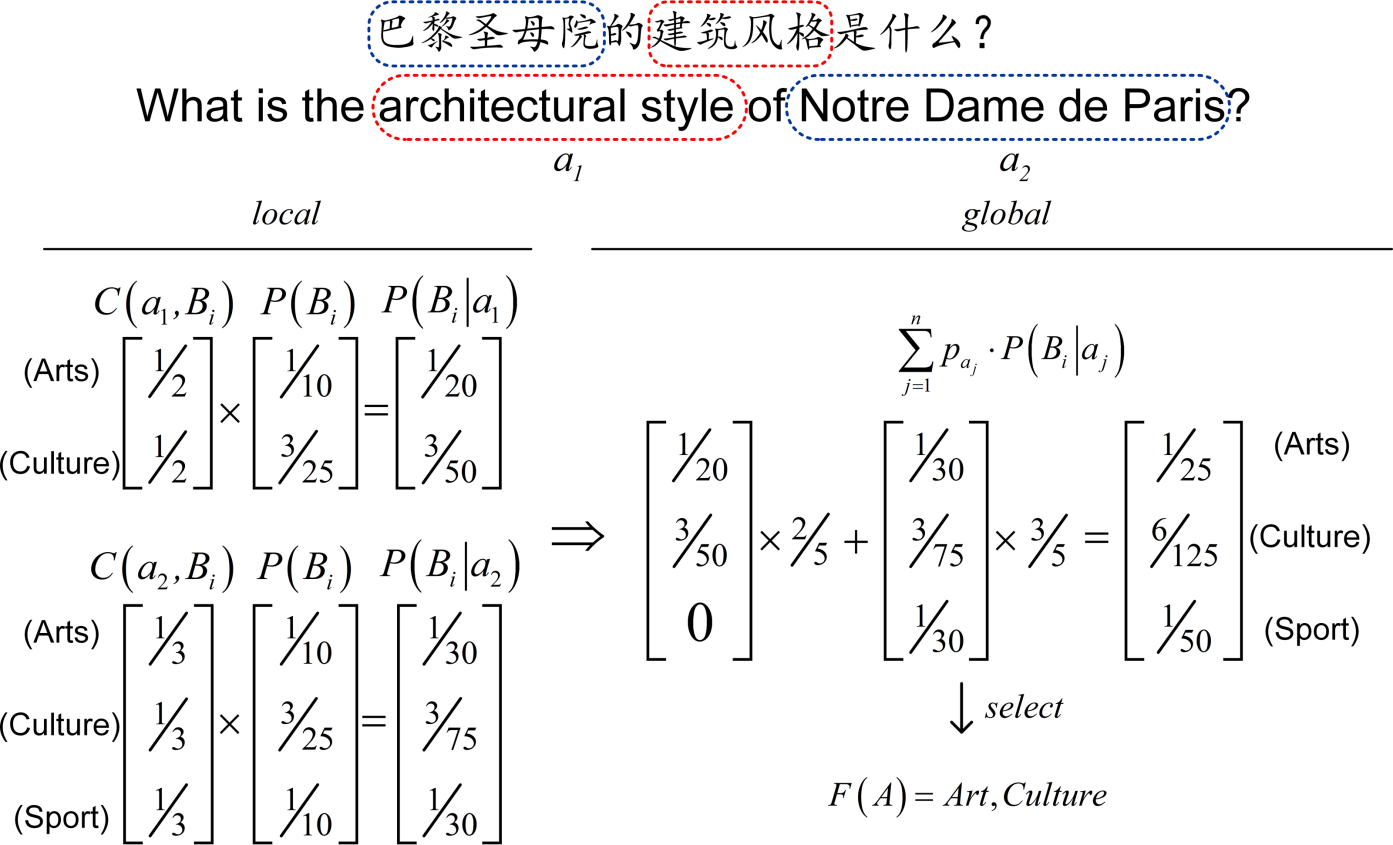
An example of our model.

Figure 3 shows an example of the DME model. For the question “What is the architectural style of Notre Dame de Paris?”, *a*_1_ and *a*_2_ is the feature terms are obtained by matching the question with dictionaries. As shown in the Fig. 3, the standard deviation *σ* be calculated as 0.012. After calculating ${s}_{1} \left( 0.008\leq \sigma \right) $ and ${s}_{2} \left( 0.02\gt \sigma \right) $, the domain of the query $F \left( A \right) $ is Art and Culture. We can similarly obtain the entity domain by analyzing the relations around it *via* DME.

**Figure 4:**
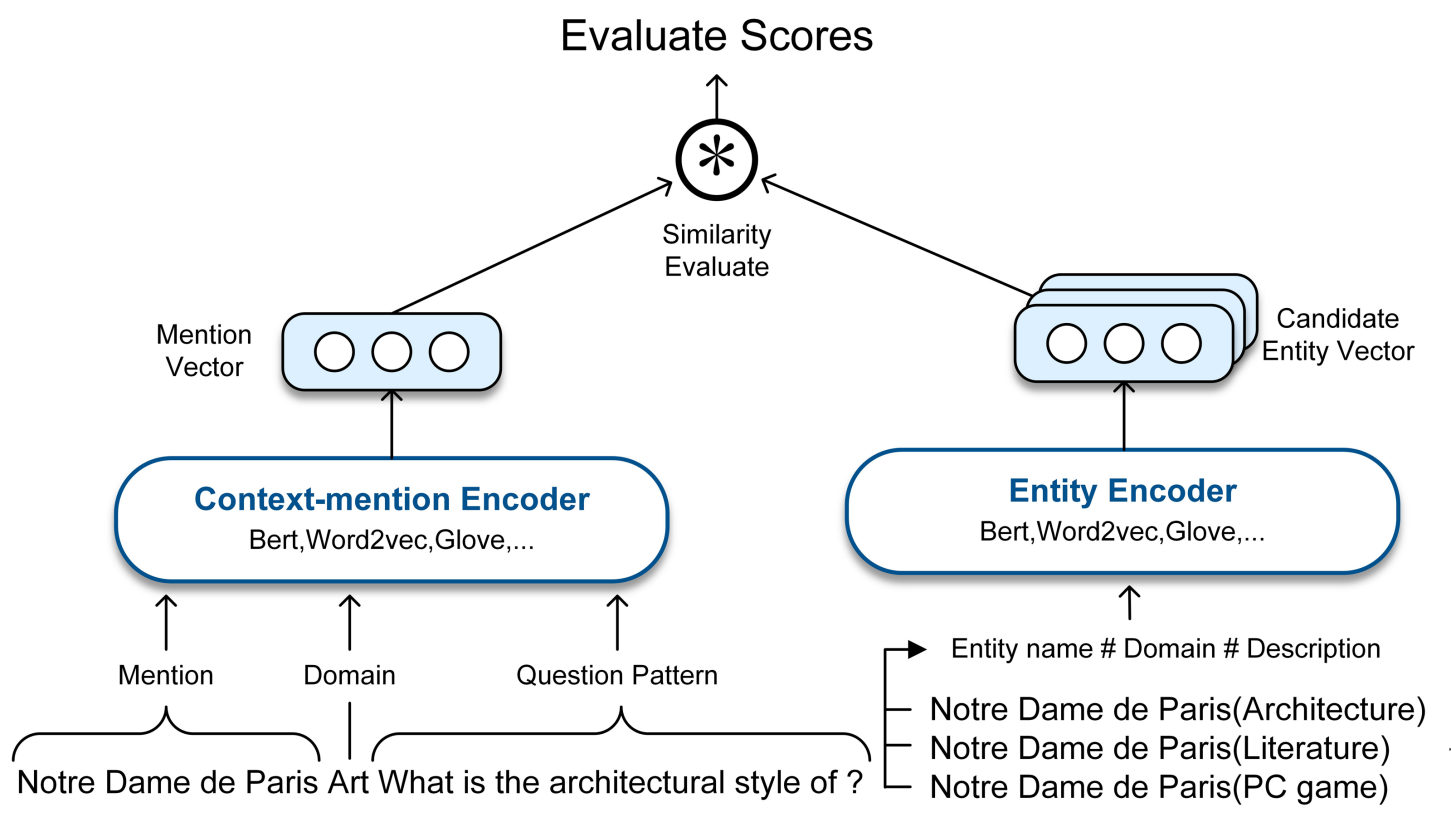
The architecture of the candidate entities ranking model.

After obtaining the domain information of both mentions and entities as external information, we used it as a part of the input for training. In training, we represented each pair of a mention *m* and an entity *v*as a sequence in the format: “*mention#field#questionpattern*”and “*entityname#field#description*”, where *question pattern* is the question string with the *mention* removed, and *description* is the text acquired by the one-hop relations of the entity *v*. Figure 4 shows our candidate entities ranking model, where Bert, Word2vec, Glove and KgCLUE baseline model are used as the baseline models for mention and entity encoding. To demonstrate the effect of our approach, we represented mentions and entities with the existing PLMs. At last, we ranked the candidate entities by calculating the cosine correlation between mention and candidate entities.

### Negative sampling approach

We regarded a correctly corresponding mention–entity pair as a positive sample and an incorrectly corresponding pair as a negative sample. Semantic matching can be regard as a binary classification task that is assigned while training. We considered that a better model could be trained if we created a set of high-quality negative samples of mention–entity pairs. The conventional approach to creating negative samples that uses random or normal distributions is blind and may even be deleterious in model training because it neglects any information that a sample takes (Cai et al., 2020).

An analysis of samples that were misclassified by the baseline models showed two typical situations: some pairs that are approximately semantically identical, such as “NBA” and “NCAA”, were identified as identical; the another is the surface form similar “University of York” (in the United Kingdom) and “York university” (in Toronto, Canada). Most approaches, for this situation, prefer negative sampling to enrich the training datasets and guide the learning process. Inspired by the representational approaches (Rao, He & Lin, 2016; Zhang et al., 2019), we considered both semantics and surface form for improve the quality of negative sampling.

For selecting the alternative options for our negative sampling strategy, we enumerated several traditional and popular approaches for measurement of proximity: Minkowski distance, Edit distance and Cosine distance. Minkowski distance and special cases based on it, such as Euclidean distance, Hamming distance and Chebyshev distance, are not friendly to high-dimensional vectors due to the low interpretability of physical meaning (Wang & Dong, 2020). Edit distance focuses on the morphological differences between the strings, which is sensitive to the surface dissimilarity. Cosine distance concentrates more on the difference of direction of vectors rather than absolute values. Compared with Minkowski distance, the Cosine distance emerge a better performance in proximity measurement of high-dimensional vectors. We thus prefer utilizing the Edit distance and Cosine distance in our negative sampling strategy.

For semantic matching, we used the pre-trained BERT model to encode the entities with descriptions and measured proximity by cosine distance:

(8) \begin{eqnarray*}similarity=\mathit{cos} \left( \theta \right) = \frac{A\cdot B}{ \left\| A \right\| \cdot \left\| B \right\| } = \frac{\sum _{i=1}^{n}{A}_{i}\times {B}_{i}}{\sqrt{\sum _{i=1}^{n}{ \left( {A}_{i} \right) }^{2}}\times \sqrt{\sum _{i=1}^{n}{ \left( {B}_{i} \right) }^{2}}} .\end{eqnarray*}

For surface form matching, we used Levenshtein distance ratio, a classical algorithm for Edit distance calculation:

(9) \begin{eqnarray*}r= \left( sum-ldist \right) /sum\end{eqnarray*}

where *sum* is the overall length of *str*1 and *str*2, and *ldist* is the edit distance.

Intuitively, a good negative sample will have a balance between obviously similar and dissimilar items. We finally combine surface form and semantics in a rule for negative sample selection:

(10) \begin{eqnarray*} \left\{ \begin{array}{@{}l@{}} \displaystyle 1,if\alpha \lt Le{v}_{\overline{e},{e}_{i}}\lt \beta ~and~\gamma \lt \mathit{Cos}~Sim \left( \overline{e},{e}_{i} \right) \lt \delta \\ \displaystyle 0, otherwise \end{array} \right. \end{eqnarray*}

where $Le{v}_{\overline{e},{e}_{i}}$ and $\mathit{Cos}Sim \left( \overline{e},{e}_{i} \right) $ are respectively the surface form and semantic similarities between $\overline{e}$ in positive mention-entity pairs and entities *e*_*i*_ in KB. The parameters *α*, *β*, *γ*, *δ* are hyper parameters for tuning; the optimal values were respectively 0.9, 0.5, 0.8 and 0.6.

## Experiment

### Data preparation

CLUE is one of the most authoritative benchmarks in the field of Chinese language understanding. KgCLUE (Xu et al., 2020) is a carefully designed Chinese KBQA benchmark based on CLUE and combining the characteristics and recent development trends of KBQA, which contains a KB, a QA dataset and several baseline model for different tasks. We chose the dataset provided by the large Chinese open source KgCLUE as the basic data source for our experiment; it contains 18,000 training pairs, 2,000 valid pairs, 2,000 test pairs and an original KB. To test the adaptability of our method we selected a dataset similar but owns more noise than KgCLUE, named NLPCC2016, which contains 14,609 training pairs and 9,870 test pairs. We chose KBQA datasets rather than entity linking datasets for two reasons: our approach to entity linking was directed towards KBQA; and most entity linking datasets are created from anchor text that targets web pages, which may be unrealistic for practical use.

### Baseline models

In order to test whether our approach can improve the ability of entity linking models without structurally changing the models, we chose three widely used PLMs for mention and entity encoding: BERT (Devlin et al., 2019), Word2vec (Mikolov et al., 2013) and Glove (Pennington, Socher & Manning, 2014). We also experiment with KgCLUE provided baseline model for similarity calculation that was based on RoBERTa. In the experiment, we fine-tuned BERT and the KgCLUE baseline model and use the original Word2vec and Glove algorithm to generate variety representations.

### Semantic matching results

We regard semantic matching as a binary classification task and test the effectiveness of our approach *via* it. To verify that the DME model processed data can be applied to multiple models, the BERT, KgCLUE provided baseline model, Word2vec and Glove are chosen as the baseline model. Table 3 shows the Accuracy and the F1 scores of three PLMs and KgCLUE provided baseline model with KgCLUE datasets. Fine-tuned BERT performed better than pretrained Word2vec and Glove. Compared with KgCLUE baseline model, fine-tuned BERT with DME-processed data increased by about 7% in accuracy and F1 scores. Obviously, the performance of these models has been improved to varying degrees with DME-processed data.

In addition, the KgCLUE and NLPCC2016 were chosen as the test datasets for adaptability of DME. Table 4 shows that the DME is suitable for different datasets and improved the accuracy and F1 score obviously.

**Figure 5:**
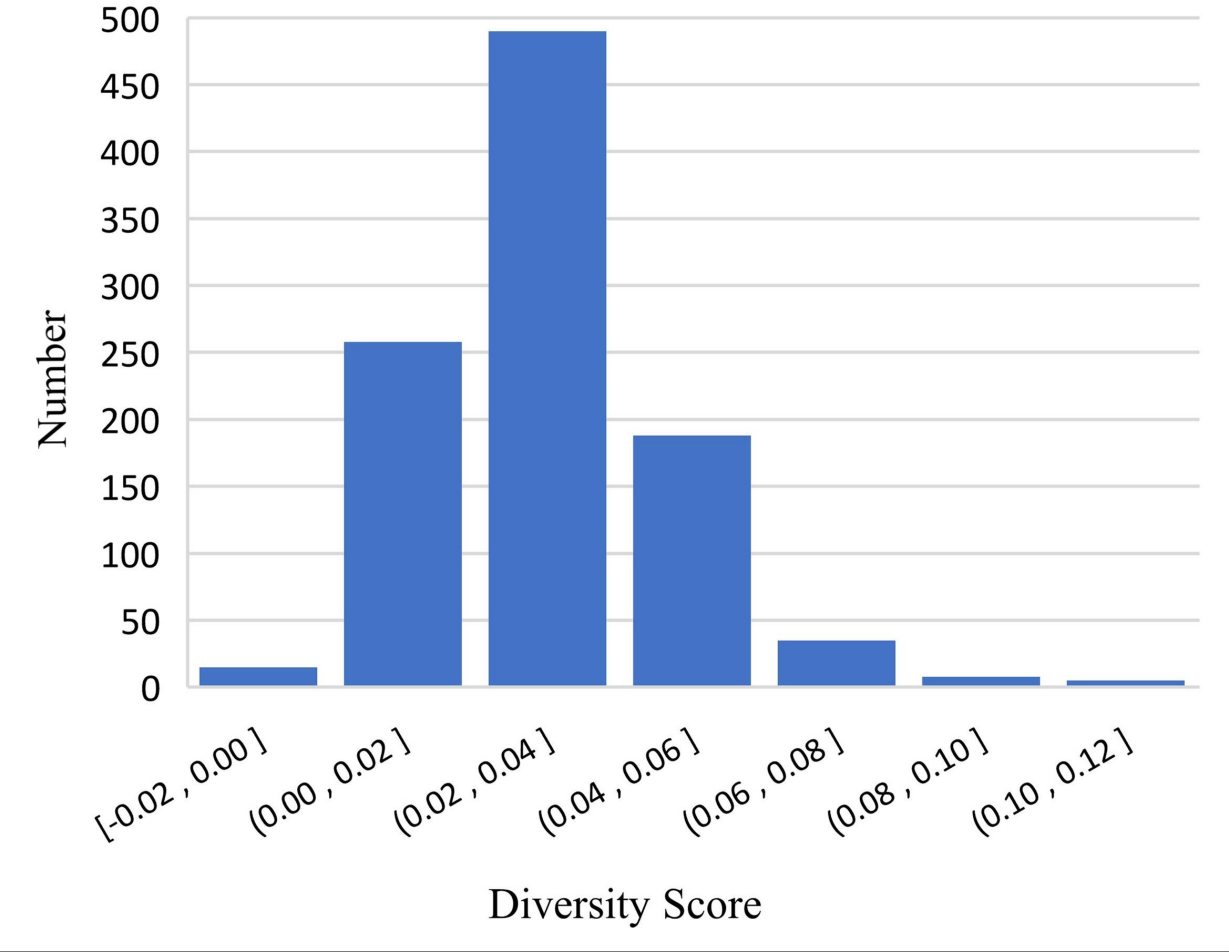
Differences in positive samples before and after DME processing.

**Figure 6:**
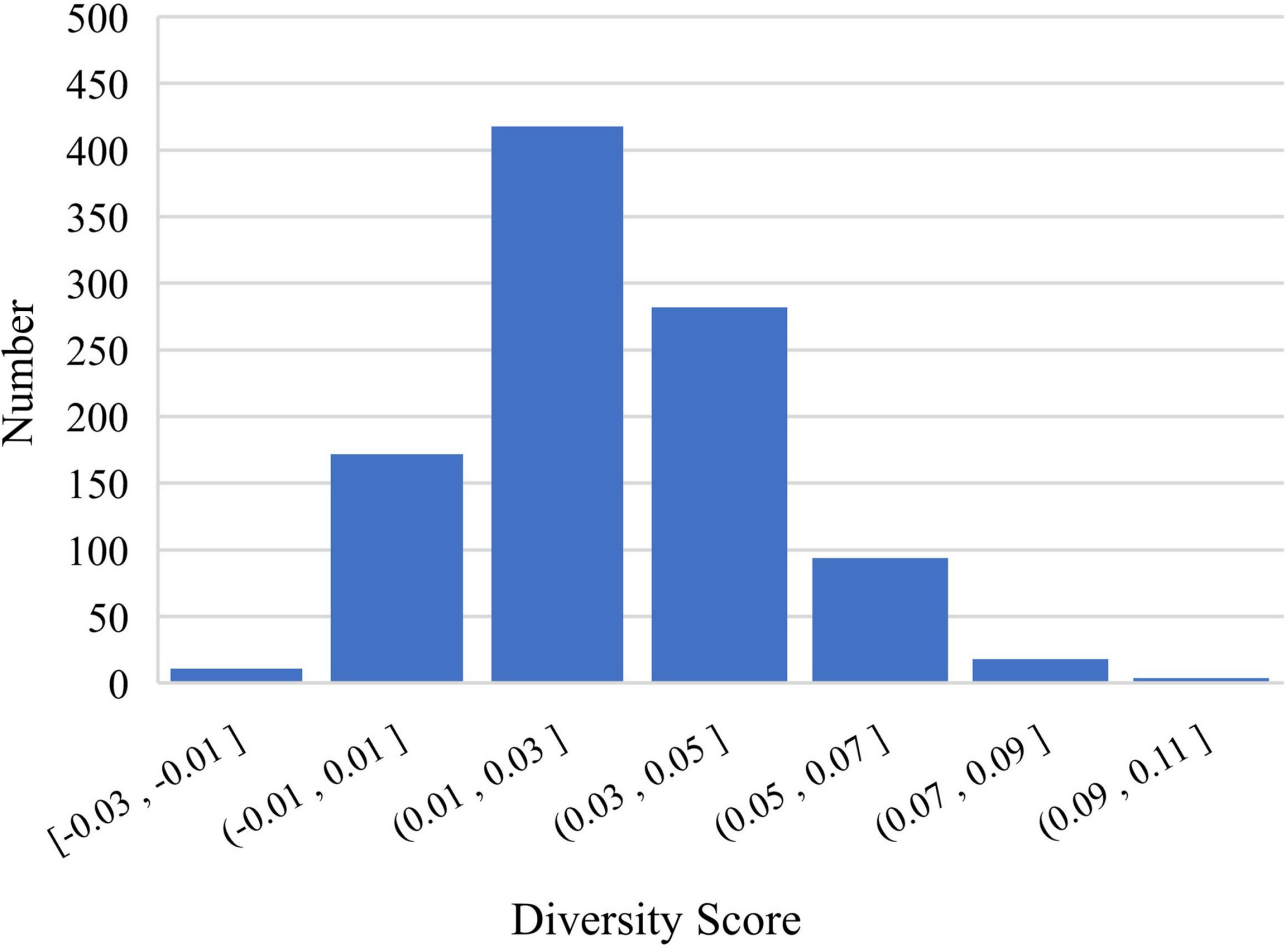
Differences in negative samples before and after DME processing.

After analyzing the results, we conjectured that DME explicitly enriches the feature dimension of the data in a manner that increases the diversity of mentions and entity representation. To verify this conjecture, we randomly sampled 1,000 positive pairs and 1,000 negative pairs for an experiment and designed a diversity score *F*_*t*_ to quantify the diversity:

(11) \begin{eqnarray*}{F}_{t}= \left\{ \begin{array}{@{}l@{}} \displaystyle {S}_{with\,~\text{domain}\,~\text{information}}-{S}_{without\,~\text{domain}\,~\text{information}}, positive~samples\\ \displaystyle {S}_{without\,~\text{domain}\,~\text{information}}-{S}_{with\,~\text{domain}\,~\text{information}}, negative~samples \end{array} \right. \end{eqnarray*}

where *S* is the cosine similarity with or without supplementary domain information. Figures 5 and 6 show the results of the experiment, which verified our conjecture.

**Table 3:** Performance of all the models using the KgCLUE dataset with and without domain information as indicated by accuracy and F1 scores.

Models	Accuracy	F1 scores
Bert (Original datasets)	84.61%	84.14%
Bert (DME-processed datasets)	94.03%	95.23%
KgCLUE baseline model	87.20%	88.52%
Glove (Original datasets)	62.08%	65.26%
Glove (DME-processed datasets)	66.40%	67.35%
Word2Vec (Original datasets)	65.84%	65.96%
Word2Vec (DME-processed datasets)	70.71%	70.57%

**Table 4:** Performance of BERT using the KgCLUE and NLPCC2016 with and without DME treating as indicated by accuracy and F1 score.

	KgCLUE		NLPCC2016	
	Accuracy	F1 score	Accuracy	F1 score
BERT with original dataset	84.61%	85.28%	84.76%	84.07%
BERT with DME-processed datasets	94.03%	95.23%	86.35%	86.50%

### Negative sampling results

To verify the effectiveness of our negative sampling strategy, with the DME-processed KgCLUE datasets, we compared our strategy with the random negative sampling strategy. Table 5 shows that our strategy produced a better result, which shows that hard negative samples better trained the model in terms of extending the decision boundary. In time overhead, our proposed method reduces 36.73% compared to random sampling. Table 6 shows the accuracy for different numbers of negative samples, one positive sample with two negative samples gets the highest measurement scores.

**Table 5:** Test classification accuracies with different negative sampling strategies.

Method	Accuracy	F1	Time cost
Without negative sampling	84.61%	84.14%	–
Random	86.33%	85.27%	294′
Entity replacement with wordform and semantic	94.03%	95.23%	186′

**Table 6:** Test classification accuracies with different number of negative samples.

Num	1	2	3	4	6	8	10	12
Acc	92.36%	94.01%	93.85%	93.67%	93.76%	93.79%	93.76%	93.02%
Recall	89.27%	91.64%	91.08%	90.53%	90.65%	90.80%	90.92%	89.55%
F1	93.14%	94.95%	94.81%	94.71%	94.80%	94.79%	94.76%	94.20%

### Ablation experiment results

To verify each individual component that we put forward to improve the entity linking task, an ablation experiment was proposed. In our research, DME, random negative sampling strategies and negative sampling strategies combining wordform and semantic are all approaches that can affect the quality of the dataset and, furthermore, the performance of the model. We therefore use these approaches to process the KgCLUE dataset separately or jointly and fine-tune a BERT model with different datasets. Table 7 shows the impact of each approach and their combination on the entity linking task.

**Table 7:** The impact of each approach and their combination on the entity linking task with the KgCLUE dataset.

BERT	DME	Random	Semantic	Wordform	Accuracy	F1
√					84.61%	84.14%
√	√				88.90%	91.83%
√		√			81.68%	82.93%
√			√		90.32%	88.80%
√				√	88.34%	87.75%
√			√	√	91.90%	90.48%
√	√		√	√	94.03%	95.23%

### Discussion

In this research, we increased the robustness of EL in the model without changing the model structure by designing a data-centric model, DME, to mine domain information for short text. Besides, an innovative negative sampling approach that considering both surface form and semantic information was proposed to generate high quality negative samples. Overall, the three main parts of experiments support the methods well. In this subsection we will have a discussion with the results above.

We implemented a set of experiments to test the effectiveness and transferable ability of DME. Table 3 shows the Accuracy and the F1 scores of three PLMs and KgCLUE provided baseline model with KgCLUE datasets. Obviously, the performance of these models has been improved to varying degrees with DME-processed data. The results supported our intuition that DME-processed data can improve model performance without necessitating structural change to the original models. Table 4 shows that the DME is suitable for different datasets. It improved the accuracy and F1 score clearly. After analyzing the results, we conjectured that DME explicitly enriches the feature dimension of the data in a manner that increases the diversity of mentions and entity representation. Figures 5 and 6 verified the conjecture. Influenced by the errors when DME recognizing the domain of a mention or an entity, some of the *F*_*t*_ are negative numbers, which will be improved by optimizing the dictionaries.

The limited datasets and models are bounded in testing and verifying the transferable ability of our approach. The core concept of DME is to improve the performance of the model by enhancing the data quality. In fact, we usually preprocess data when training models, which is consistent with the concept of “data centric”. The reason is that we believe that better datasets can advance model training. So, we believe that DME has outstanding portability in theory, that is, it can be applied to most datasets. In the meantime, it can be effective for stronger models than BERT.

As to the negative sampling strategy that we proposed, there are two experiments raised to verify the effectiveness. Table 5 shows that our strategy produced a better result, which shows that hard negative samples better trained the model in terms of extending the decision boundary. As to the time cost, our method is much lower than random sampling. This is because a good negative sampling strategy is able to reduce the time complexity of gradient descent when fine-tuning the BERT. Sampling randomly may introduce wrongly labeled mention-entity pairs unpredictability that caused the limited improvement and higher time overhead. The results of the experiment also verified the conclusion that we proposed in the ‘Negative sampling approach’ section. Table 6 shows the accuracy for different numbers of negative samples, we inferred that one positive sample with two negative samples can optimally train the model.

In the ablation experiment part, Table 7 shows the impact of each approach and their combination on the entity linking task. By analyzing the experimental results, we may draw the following conclusions: (1) Our proposed DME model can effectively improve the performance of entity linking tasks; (2) The random sampling process will generate some false negative samples, which will make adverse impact on the model training; (3) In contrast, considering semantic information is more conducive to high-quality negative sampling than considering wordform information; (4) The combination of our proposed DME model and negative sampling strategy can achieve better results on entity linking task.

## Conclusions

In this article, we built a domain dictionary and then proposed a lightweight and effective data-centric model, DME, to mine and explicitly express domain information relating to mention and entity. In addition, we proposed a negative sampling strategy that considering both semantic and wordform information of the text. According to the experimental observation, the quantity and quality of negative samples can affect the performance of an entity linking model. Also, the combination of our proposed DME model and negative sampling strategy can achieve better results on entity linking task. The future work will be concentrated on three directions: First, improving the quality of domain dictionaries by expert inspection to increase the accuracy of DME; second, proving the effectiveness of our proposed method on some recently proposed advanced models, third, extending the DME to more tasks such as the text classification and search engine.

##  Supplemental Information

10.7717/peerj-cs.1233/supp-1 Supplemental Information 1 The code of the DME model DME is a domain information sensitive model which is able to mine and explicitly express the domain information of a string.

10.7717/peerj-cs.1233/supp-2 Supplemental Information 2 The code used to transform the original data to train needed data

10.7717/peerj-cs.1233/supp-3 Supplemental Information 3 The code used to fine-tune a BERT model based on our dataset

10.7717/peerj-cs.1233/supp-4 Supplemental Information 4 The results of diversity score of positive pairs

10.7717/peerj-cs.1233/supp-5 Supplemental Information 5 The results of diversity score of negative pairs

## Additional Information and Declarations

### Data Availability

The following information was supplied regarding data availability: The code and data are available in the Supplemental Files.
